# Cell-to-Cell Spread of Retroviruses

**DOI:** 10.3390/v2061306

**Published:** 2010-06-10

**Authors:** Quentin J. Sattentau

**Affiliations:** The Sir William Dunn School of Pathology, The University of Oxford, South Parks Road, Oxford OX13RE, UK; E-Mail: quentin.sattentau@path.ox.ac.uk; Tel.: +44 1865 275511; Fax: +44 1865 275515

**Keywords:** HIV-1, virological synapse, CD4^+^ T cell, macrophage, dendritic cell, membrane fusion, endocytosis, immune evasion

## Abstract

Viruses from several families use direct cell-to-cell infection to disseminate between cells. Retroviruses are a relatively recent addition to this list, and appear to spread cell-to-cell by induction of multimolecular complexes termed virological synapses that assemble at the interface between infected and receptor-expressing target cells. Over the past five years, detailed insight into the cellular and molecular basis of virological synapse-mediated retroviral cell-to-cell spread has been obtained, but important questions and controversies have been raised that remain to be resolved. This review will focus on recent advances in the field with emphasis on areas in which work still needs to be done.

## 1. Introduction

Mammalian viruses have co-evolved with their hosts, and in doing so have developed elegantly adaptive mechanisms for invasion, persistence and onward transmission. Since viruses are obligate intracellular pathogens, they harness the cellular machinery to enter, replicate and exit. The classical paradigm of viral propagation by release of independent infectious virus particles that diffuse freely in extracellular fluids is robust, and for most viruses is probably the best mechanism for long-distance viral dissemination within, and between hosts. However, another mode of viral spread, somewhat neglected in recent years, has relatively recently re-emerged; that of directed movement of viruses between contacting target cells without recourse to long-distance fluid phase diffusion. Such ‘cell-to-cell’ spread was first formally demonstrated for herpesviruses. Varicella Zoster virus was efficiently passed between cells only in a cell-associated form [1], and herpes B virus resisted antibody neutralization when spreading directly between cells, but was neutralization sensitive when spreading in a cell-free mode [2]. Twenty years later, the cell-to-cell movement of rhabdoviruses across neural synapses was captured by electron microscopy [3]. Members of several virus families have adopted cell-to-cell spread by various means including: budding from infected cells and entering target cells across epithelial cell tight junctions (herpesviruses); elaborating actin-rich cellular structures that propel virions from infected cells directly into uninfected cells (poxviruses); breaking down intercellular barriers to spread by inducing limited membrane fusion between infected and uninfected cells (paramyxoviruses) [4,5]. Today the concept of directed cell-to-cell viral spread is well accepted, and for the viruses mentioned above, the cellular and molecular mechanisms are at least partially understood.

## 2. Retroviruses and Virological Synapses (VS)

Retroviruses have a relatively small genome (∼10 kb) and interact in a particularly intimate manner with their cellular hosts. Being enveloped, retroviruses are fragile and do not survive well outside of an infected cell; decay of infectivity is rapid even in tissue culture. Immunodeficiency viruses such as HIV-1 are particularly prone to infectivity decay as their envelope glycoprotein (Env) spike is non-covalently assembled and dissociates into non-functional components over time [6,7]. It is therefore important for their optimal survival and dissemination to find and infect new host cells with the minimum of delay. Many retroviruses infect immune cells, and this is central to the pathogenesis of HIV-1, HIV-2 and simian immunodeficiency virus (SIV)-induced AIDS and human T cell leukemia virus type-I (HTLV-I)-induced disease. Immune cells generally do not have inbuilt polarity, unlike epithelial cells for example [5], and so the spread of retroviruses between immune cells seemed unlikely to be mediated by the same type of mechanism adopted by herpesviruses or rhabdoviruses. Early studies highlighted the ability of HIV-1 to induce syncytium formation between infected and uninfected target cells, suggesting a potential mechanism for intercellular spread of virus similar to that described for paramyxoviruses [5]. However, more recent work revealed that this was in large part an artifact of adaptation of viruses to growth in immortalized cell lines, and restricted principally to CXCR4-using viruses [8]. Interestingly, only limited cell-cell fusion of infected and uninfected cells has been observed during retroviral cell-to-cell transfer, despite estimated infected-target cell conjugate lifespans of several hours or more [9–11]. Several studies support the concept of active suppression of HTLV-I [12] and HIV-1 [13,14]–mediated cell-cell fusion by recruitment of the regulatory tetraspans CD9, CD81 or CD82. Other mechanisms, such as exclusion of Env and/or viral receptors from regions of adhesion between infected and target cells, may also operate. What therefore might be the dominant mechanism of cell-to-cell transfer of HIV-1? Clues came from emerging evidence in the field of immunological antigen presentation, which revealed communication between immune cells via immunological synapses [15]). This model implied that both antigen-presenting cells (APC) and lymphocytes were able to co-polarize their cytoskeletons for the purpose of directed exchange of cell-surface (receptor-ligand) and soluble (cytokine) biochemical information leading to modulation of cell function. Similar CD4 [16] and adhesion molecule [17] polarization had already been observed by ourselves and others when HIV-1-infected cells contacted CD4^+^ target T cells, and this was shown to be gp120- and actin-dependent [18]. Around the same time, pivotal observations were made by the Steinman laboratory that dendritic cells (DC) were able to efficiently transfer HIV-1 to CD4^+^ T cells via adhesive conjugates [19,20]. These findings led to the concept that retrovirally-infected cells might act in a manner analogous to the immunological synapse by coordinating viral exit on the infected cell and viral receptors on the ‘target’ cell, and the term virological synapse (VS) was proposed by ourselves in 2002 to describe this macromolecular reorganization [21]. Seminal papers describing imaging of synaptic transfer of retroviruses were subsequently published, concerning HIV-1 cell-to-cell spread between DC and CD4^+^ T cells across an ‘infectious synapse’ [22] and HTLV-I [23] and HIV-1 [9] spread between T cells via VS. All papers alluded to polarization of the cell types involved: McDonald *et al.* described polarization of HIV-1 particles and viral receptors to the DC-T cell contact zone, Igakura *et al.* observed clustering of viral Gag in the infected T cell and the actin-associated adaptor molecule talin on the target cell, and Jolly *et al*. observed co-polarization of HIV-1 Env, Gag and viral receptors CD4 and CXCR4. Several papers rapidly followed that confirmed and extended these observations of VS structure and function for HIV-1 in a variety of cell types including DC, T cells and macrophages [9,10,24–28]. Later studies pointed out that HIV-1 could not only assemble single large-scale VS containing pairs of conjugated cells, but also ‘polysynapses’ containing multiple target cells [28,29].

The structure and function of retroviral VS and their relationship to immunological synapses will be reviewed elsewhere in this issue of *Viruses*, so here I will only summarize their principal molecular features.

1. A central feature of intercellular synaptic structures is the presence of an adhesive junction that is sufficiently stable to allow transfer of signals and soluble mediators between the pre- and post-synaptic cells [5,30]. In the case of the HIV-1 T cell VS, the initial cognate event that holds the interacting T cells together appears to be CD4-gp120 binding [9], and subsequent stable adhesive junctions are probably maintained by integrin-ICAM interactions [17,31,32], although others dispute a functional role for cellular adhesion molecules in this context [33].
2. The interaction of Env on the HIV-1-infected cell with CD4 on the target cell recruits filamentous actin into the synaptic zone along with more CD4, HIV-1 coreceptor (CXCR4 or CCR5) and adhesion molecules [9,31]. However, actin may be cleared from the central region of the VS, a strategy proposed to facilitate viral entry into the target cell [34].
3. There is evidence that HIV-1 may assemble at the plasma membrane of infected T cells at sites of cell-cell contact, implying polarization of cellular secretory systems towards the VS [35]. This is supported by movement of the microtubule organizing center (MTOC) and other elements of the secretory apparatus proximal to the site of cellular contact in both HTLV-I- and HIV-1-infected T cells [35–40].

The T cell VS consists of relatively large opposing surfaces of polarized infected and target cell plasma membrane containing, respectively, clustered Env and viral receptors [11,28]. However, HIV-1 has also been observed to travel along long intercellular tubular structures termed ‘membrane nanotubes’ joining infected and uninfected T cells [29,41]. Nanotubes emanating from infected and uninfected T cells appear to join at a ‘micro-synapse’ [41], which migrating virions must pass to infect the target cell. HIV-1 infection via membrane nanotubes appears to be less common than via VS [29]. Very recently, the movement of HTLV-I anchored at the infected cell surface in a meshwork of extracellular matrix (termed a ‘viral biofilm’) to uninfected T cells has been proposed as an alternative to VS-mediated transfer [42]. Whether this is a dominant mode of infectious HTLV transfer, and whether it applies to other retroviruses, remains to be seen, although this phenomenon may be related to the ‘antigen caps’ observed in unconjugated T cells infected with HIV-1 for extended periods of time [43–45]. A retrovirus that infects predominantly non-immune cells, murine leukemia virus (MLV), spreads between fibroblasts by surfing along projections emanating from the infected cell termed filopodia, anchored to the target cell by interactions between the viral glycoproteins on infected cells and viral receptor on target cells [46,47].

## 3. Retroviral transfer across VS: fusion and/or endocytosis?

**Figure 1:**
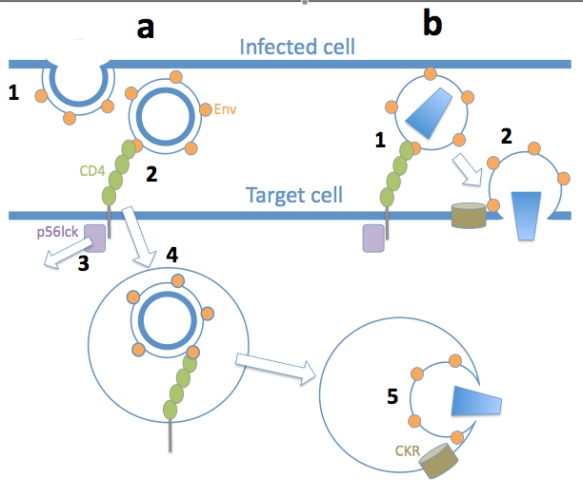
**Hypothetical mechanisms of HIV-1 uptake and entry across VS. (a)** Acutely-infected T cell VS**. 1)** HIV-1 budding from an acutely-infected T cell leads to production of immature virus particles at a VS. **2)** Fusion-incompetent HIV-1 Env within the plasma membrane, in budding virions or in budded immature virions, engages CD4 on the target cell plasma membrane leading to **3)** dissociation of p56lck from CD4 that results in **4)** endocytosis of CD4 complexed with immature HIV-1. **5)** After a time delay for virus maturation, the mature, fusion-competent virion fuses with the endosomal membrane in a CD4 and chemokine receptor (CKR)-dependent manner. **(b)** Longer-term infected T cell VS. **1)** HIV-1 budding from a longer-term infected T cell will result in mature, fusion-competent virions associated on the surface in a polarized cap. Binding of viral Env to CD4 on the target cell plasma membrane results in **2)** direct fusion of the viral envelope with the cell plasma membrane in a CD4 and CKR-dependent manner, leading to rapid infection of the target cell.

That HIV-1 can spread between T cells by directed cell-to-cell spread now appears unequivocal. However, it remains to be fully understood what proportion of synaptic transfer events results in infection of the target cell, and where in the cell most infection takes place. Several groups have reported that HIV-1 Gag moving across a VS from an infected CD4^+^ T cell may accumulate within the target cell in endosomal compartments [10,28,48] and reviewed in [49]. A proportion of the virus may then fuse from within the endosomal compartment, resulting in a delayed but potentially productive infection [50]. A similar series of events has been reported for DC to T cell spread [51]. However, the dominant uptake of viral Gag via endocytosis has not been detected in all studies. Our own laboratory has failed to observe endocytic uptake of HIV-1 across VS by confocal miscroscopy, electron microscopy or electron microscopy with tomography [9,11]. These variations in the mechanism of viral uptake have been suggested to result from the differential use of primary cells compared to immortalized cell lines [49]. In this article, Puigdomenech *et al.* hypothesize that primary cells may be inefficient for productive HIV-1 entry, resulting in endocytosis as the dominant observed mode of viral uptake leading to a gp41-mediated form of cell death in the target cells [49]. By contrast, they propose that immortalized CD4^+^ T cell lines are more permissive for HIV-1 fusion, and so endocytosis is less dominant and productive infection more prominent. Although the conclusion that primary CD4^+^ T cells are less efficient for HIV-1 entry than some immortalized CD4^+^ T cell lines is consistent with existing literature [11,52,53], the explanation does not seem to be this simple. For example, Jolly *et al.* [9] investigated primary CD4^+^ T cells as HIV-1 targets and failed to observe colocalization of transferred HIV-1 Gag (or Env) with the early endosome marker EEA1, although it should be noted that EEA1 is not a universal marker of endosomal uptake [54]. Moreover, neither in [9] nor [11] was there evidence of HIV-1 uptake into intracellular compartments of primary CD4^+^ T cells using thin section electron microscopy or electron tomography. However, others do report virus-like particles in uncharacterized vesicular structures in target primary CD4^+^ T cells [28,48], although the lack of tomographic analysis does not allow unequivocal demonstration that these are intracellular compartments rather than plasma membrane invaginations as observed in macrophages [55–57]. Another relevant experimental variable may be the chronicity of infection in the infected cell prior to analysis of infected-target cell conjugate formation [28]. Studies from our laboratory have mostly been carried out using CD4^+^ T cells (cell lines or primary cells) that are infected for up to a week to increase the proportion of infected cells going into the assay [9,11]. One consequence of this may be that during culture, infected cells form contacts with uninfected cells, inducing VS and potentially releasing virus particles onto the cell surface in the form of polarized viral assembly ‘caps’ [43–45] that may be analogous to HTLV-1 biofilms [42]. If such longer-term infected cells have mature infectious HIV-1 attached to the cell surface at these polar caps then subsequent contact with uninfected CD4^+^ T cells may allow rapid infection of the target cell by direct-virion fusion with the target cell plasma membrane (Figure 1). By contrast, acutely-infected CD4^+^ T cells may not have made VS within the culture and therefore polarization and budding may occur for the first time during conjugate analysis for VS formation. Under these conditions, virus freshly-released from the infected cell into the VS may be largely immature, and therefore fusion-inefficient [58,59]. Such immature virus may be taken up into the cell by an endocytic mechanism, and may then fuse from within the endosomal compartment when maturation is complete (Figure 1). This hypothesis would be in accord with the method of preparation of infected cells and the data presented in [28], and the trypsinization of infected cells to remove cell surface-associated virus prior to co-culture with target cells in [60], but not with the longer-term infections used in other studies reporting on viral endocytosis across VS [48]. Thus, the discrepant observations relating to the form of viral transfer and the outcome of infection across VS may be more complex still than considered here. Moreover, questions remain regarding the kinetics of viral maturation into a fusion-efficient state: this process requires more quantitative analysis. A final variable that needs to be taken into account is cell-cell fusion triggered in co-cultures of infected and uninfected T cells. Most of the recent studies on VS-mediated spread of HIV-1 in culture do not observe significant levels of fusion between HIV-1-infected and uninfected target cells [9,28,53]. By contrast, Ruggiero *et al.* [60] propose that cell-cell fusion might be an important component of cell-to-cell HIV-1 spread, although their data appear to be based primarily on immortalized T cell lines, which are thought to be inherently more fusogenic than primary T cells.

Although we do not yet have an explanation backed up by experimental evidence for the discrepant results relating to prevalence of HIV-1 uptake by endocytosis after cell-to-cell transfer of HIV-1, we propose that the mechanism of uptake of virus by the target cell, which will relate directly to the method of detection of viral transfer, is of secondary importance to the functional outcome of the transfer. For example, Gag labeling as a method of detection of viral transfer, although important for live- and fixed-cell imaging, is a technique that on its own cannot discriminate between non-infectious and infectious transfer events. Given the generally accepted feature of relatively low infectivity:particle ratios in retroviruses, it might be expected that most virions transferred to a target cell by cell-to-cell spread will fail to infect, leading to accumulations of ‘dead’ virus within the VS, adhering to the target cell surface, and potentially taken up into the target cell by various endocytic mechanisms. However, cell-to-cell spread across the VS would tend to increase the infectivity:particle ratio by virtue of the rapidity of viral transfer across the VS and the concomitant clustering of adhesion molecules and viral entry receptors on the opposing target cell [61]. Whether or not fusion from within endosomes is a more permissive route of viral infection of CD4^+^ T cells than fusion at the cell surface [50,60,62], the high multiplicity of infection imparted onto the target cell by VS-mediated spread will undoubtedly result in efficient infection by one or both of these routes. We propose, therefore, that the simplest manner to overcome discrepant results concerning the uptake of virus via VS is to use assays that measure outcomes directly relevant to infection of the target cell. These include the use of: i) recombinant viruses containing early gene reporter constructs such as GFP [28]; ii) cell lines that activate a reporter construct upon HIV-1 infection [28,60]; iii) single-cycle replication-dependent reporter vectors [63]; iv) qPCR to measure de-novo reverse transcribed viral DNA [11,31,44]; v) detection of de-novo synthesized, inhibitor-sensitive Gag production in the target cell [53].

## 4. VS and evasion of entry inhibition

**Figure 2:**
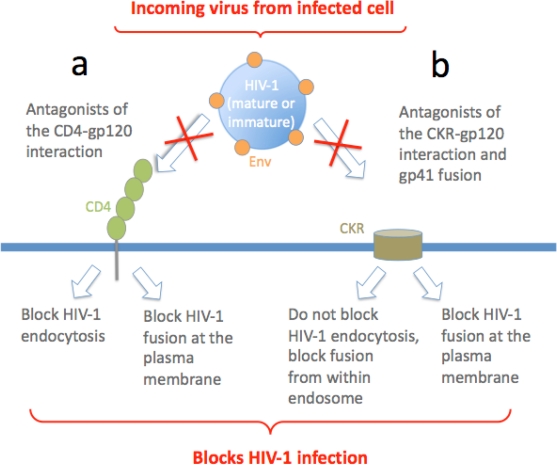
**Inhibition of HIV-1 uptake and entry across VS. (a)** HIV-1 (mature or immature) engaging CD4 may trigger either fusion at the plasma membrane (mature virus) or endocytosis (immature virus): both of these events are prevented by blocking the CD4-gp120 interaction, and so HIV-1 cell-to-cell transfer and infection are inhibited. **(b)** HIV-1 (immature) engaging CD4 and the CKR coreceptor is unable to efficiently activate gp41-mediated fusion, and so virus is internalized by CD4-mediated endocytosis, and antagonists of gp120-CKR interaction or gp41-mediated fusion do not prevent endocytosis. However once in the endosome HIV-1 matures into a fusion-efficient form and gp120-CKR or gp41 antagonists prevent HIV-1 fusion with the endosomal membrane, preventing HIV-1 cell-to-cell infection. HIV-1 (mature) engaging the CKR triggers rapid gp41-induced plasma membrane fusion and both of these events can be blocked by antagonists, preventing cell-to-cell infection.

Cell-to-cell spread of herpesviruses was first defined by its resistance to neutralizing antibodies (NAb) of a spreading infection in culture compared to the sensitivity of fluid-phase virion dissemination [2,64]. Neural synapses are though to be sealed from the outside environment by virtue of a tight adhesive junction [30], allowing rhabdoviruses to spread directionally by this route [65] presumably avoiding attack by NAb. Poxviruses [66] and hepatitis C virus [67] have also been proposed to use cell-to-cell spread as a means to escape antibody neutralization. It has therefore been speculated that retroviruses might use VS to evade attack by NAb and other types of entry inhibitor [5,68]. At present, it is unclear whether this is the case, since confusion exists in the field as a result of studies reporting discordant results. There is general agreement that inhibitors of the CD4-gp120 interaction inhibit VS-mediated viral transfer independent of the system used to detect HIV-1 in the target cell [9,10,33,48,60]. By contrast, some of the same groups reporting on Gag transfer between cells using antagonists of events proximal to viral fusion, including NAb to gp41 [10] and HIV-1 coreceptor antagonists [10,69], failed to inhibit Gag transfer but did inhibit infection [70]. At one level these data can be reconciled by the explanation that the CD4-gp120 interaction is required to trigger the stable association of infected-target cell conjugates to form VS, without which no virus transfer could take place [9,48]. In this respect all groups agree that both endocytosis and infection are prevented by inhibitors of the CD4-gp120 interaction [49], and this is consistent with earlier work investigating cell-free viral endocytosis into T cells [62]. With regard to a central role of the Env-CD4 interaction in endocytosis of HIV-1 by the target cell, it is interesting to invoke the endocytic capacity of CD4 conferred by motifs within its cytoplasmic tail [71]. CD4 endocytosis can be triggered by gp120 engagement that dissociates the cytoplasmic tyrosine kinase p56lck [72], liberating CD4 from the endocytosis inhibitory activity of p56lck [73] (Figure 1). However, Blanco and colleagues observed that the cytoplasmic tail of CD4 was not required for HIV-1 uptake by a T cell line, implying that a non-classical mode of viral endocytosis may be operating [48]. Because there is limited time for the virus to mature during VS-mediated transfer to the target cell, this mode of HIV-1 spread would be expected to result in higher levels of immature virus transferring to the target cell than during cell-free infection, and therefore a higher frequency of endocytic uptake of virus [28]. The apparently conflicting data with regard to post-CD4 entry requirements for virus transferred across VS can be reconciled by considering whether the assay used to measure viral transfer detects viral endocytosis or infection as an endpoint (Figure 2). Coreceptor and fusion antagonists fail to interfere with viral transfer across VS mediated by endocytic uptake of virions, since neither the chemokine receptors CCR5 and CXCR4 nor the viral fusion apparatus (gp41) are required for this route of uptake [10,48,70]. By contrast, when viral infectivity is measured using assays of viral reverse transcription, integration or proviral transcription, all inhibitors of viral entry are effective [11,28,49,60,70]. This may be because inhibitor bound to neutralized virus or bound to viral coreceptors will prevent both direct viral fusion at the plasma membrane and membrane fusion within the endosome, and will exert its effect as the virus matures and becomes infectious (Figure 2). Indeed, we observe that when events proximal to HIV-1 infection of target cells are assayed for, inhibitors of all stages of viral entry including NAb interfere with cell-free and cell-to-cell spread with approximate equivalence, regardless of mode of action or molecular weight [11]. An interesting exception to this rule was reported by Hubner *et al.* [28], who failed to block cell-to-cell spread of HIV-1 with a patient antiserum capable of neutralizing cell-free virus. Whether this represents a qualitatively differential effect of antibody on the two modes of viral transfer, or whether this antiserum contains HIV-1 inhibitory activity of an unusual nature, remains to be seen.

**Figure 3:**
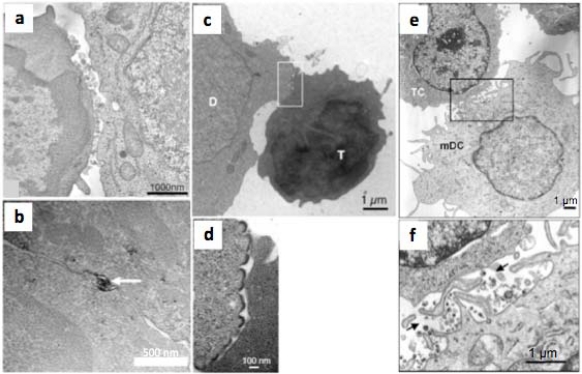
**Heterogeneity of retroviral VS. (a)** Single 2 nm thick digital slice taken from [9]. HIV-1 mature particles are seen at the interface of an HIV-1-infected T cell line (right cell) and a primary CD4^+^ T cell (left cell). The cells are loosely apposed with few points of adhesion and relatively large (>100 nm) intracellular gaps. **(b)** Thin section of an HTLV-1 VS taken from [40], showing an HTLV-I particle immunostained for Gag at the interface between a chronically-infected T cell line (top) and an uninfected primary CD4^+^ T cell (bottom). The two cells have an extensive area of closely-apposed plasma membrane. **(c)** A VS formed between an acutely-infected T cell line (donor cell, D) and a primary CD4^+^ T cell target cell (T) from [28]. **(d)** A higher magnification of the boxed image in **(c)** showing budding structures at the intracellular interface. **(e)** A VS formed between a mature DC pulsed with inactivated HIV-1 and a primary CD4^+^ T cell, showing loose contacts between the cells and multiple filopodia-like structures [75]. **(f)** A higher magnification of the boxed area in **(e)** showing filopodia and gaps containing virus-like particles between the cells, some of which are labeled with arrows.

These considerations of entry inhibitor sensitivity apply so far only to transfer of HIV-1 between T cells: relative permissivity to entry inhibition may not be the case for all types of VS, since resistance of DC-T cell viral spread to antibody-mediated inhibition has been reported [74]. Structurally, the macrophage-T cell VS appears to have broader regions of tightly apposed membrane than the T cell-T cell VS [26], although this contrasts with the apparently looser structures visualized for DC-T cell VS [75] (Figure 3). However, the conjugates imaged in [26] were derived from HIV-1-infected macrophages whereas the DCs imaged in [75] were derived from DCs pulsed with inactivated HIV-1, and thus may not be directly comparable. The T cell VS associated with HTLV-I spread [40] appears to be structurally distinct from that observed for HIV-1 [9,11], with a relatively large surface of closely apposed membrane containing occasional ‘pockets’ of virus-like HTLV-I particles [40], which may shield the virus from NAb access (Figure 3). However it is difficult to make general conclusions based upon the currently available electron microscopic data, as they are sparse and derived from different cell types under different conditions.

## 5. Conclusions and perspectives

The concept of retroviral cell-to-cell spread across VS is now well established, and its relevance for *in vivo* viral dissemination seems certain. Rapid progress in the field has provided a wealth of information about the structure and function of VS, but uncertainties remain concerning the mode of uptake of virus by the target cell and its susceptibility to inhibitors of the fusion cascade. Some of these uncertainties have been raised here, and will be experimentally addressed over the next few years. Probably the most urgent area for further research relates to the question of HIV-1 endocytosis: accumulating data suggest that at least in certain immortalized cell types with some viral isolates, viral fusion from within endosomes may be the principal pathway for cell-free virus [62,76] rather than fusion at the plasma membrane, the dominant paradigm for the past 20 years [77,78]. Resolution of this controversy will help understand discrepancies in data concerning HIV-1 entry and its inhibition from a variety of laboratories. A second area relates to the cell-type dependence of mechanisms of HIV-1 cell-to-cell transfer, and whether there are, or are not, qualitative differences in the type of VS formed. More electron microscopic imaging and tomographic reconstruction of synaptic interfaces would be invaluable. At present, the closest approximation of HIV-1 synaptic spread *in vivo* is static snapshots of HIV-1 infectious foci in intact lymphoid tissue [79–81] associated with mathematical models of cell-to-cell spread [82–84]. A third area of research, therefore, that would add enormously to our confidence in the *in vivo* relevance of this phenomenon would be intravital imaging of HIV-1 cell-to-cell spread in intact secondary lymphoid tissue, as has been carried out for other pathogen-immune cell interactions [85,86].
